# Case of Axillary Aneurism for Which the Subclavian Artery Was Tied; with an Inquiry into the Nature and History of That Affection

**Published:** 1841-06

**Authors:** S. D. Gross

**Affiliations:** Professor of Surgery in the Louisville Medical Institute


### WESTERN JOURNAL

OF

### MEDICINE AND SURGERY.

JUNE, 1841.

Art. 1.—*Case of Axillary Aneurism for which the Subclavian Artery was Tied; with an Inquiry into the Nature and History of that Affection.* By S. D. Gross, M. D. Professor of Surgery in the Louisville Medical Institute.

Daniel Monday, a free negro, the father of several children, of a stout muscular frame, and temperate habits, 36 years of age, came under my care on Saturday, February 13th., 1841, on account of an aneurism of the right axillary artery. He was an industrious, hard-working man, a brick-maker by occupation, and had always enjoyed excellent health until within the last few months. On examination, I discovered a circumscribed, pulsating tumor underneath the pectoral muscle, extending from the clavicle, which it had thrown considerably upwards, into the axilla, on the one hand, and down towards the cartilage of the fourth rib, on the other. It was of an irregular, conical shape, and about the size of a very large fist, measuring fully four inches at its base in one direction by three and a half in the other. In its feel it was very tense, as well as inelastic, and the pulsation was so distinct that it could be seen at the distance of some feet from the patient. Pressure, however firmly applied, produced no sensible effect upon it; and the blood rushed into it with a loud whizzing noise, or "bruit de soufflet." Owing to the elevation of the clavicle it was impossible to compress the subclavian artery so as to arrest the pulsation in the swelling, or even diminish the flow of blood through it. The whole limb, from the top of the shoulder to the extremities of the fingers, was benumbed, painful, and almost deprived of the power of motion; the swelling, however, was slight, and not at all œdematous in its character: the temperature was also good. Severe suffering was likewise experienced in the site of the tumor and in the lower part of the neck, from the compression, doubtless, of the axillary plexus of nerves. The pectoral muscle was stretched to the greatest extent; and the patient constantly inclined his head towards the affected side, keeping the elbow nearly at a right angle, and supporting it carefully with the opposite hand, to prevent tension of the swelling. The pulse at the wrist was nearly as distinct as in the other limb, but it now and then intermitted. For the last four weeks the pain was almost incessant; it was particularly severe at the chest and shoulder, and had become so agonizing, of late, as to deprive him, in great measure, of sleep, or even prevent him from lying down. His appetite was also much impaired, and his countenance was expressive of the deepest distress.

On questioning him as to the origin of his disease, he stated that last Christmas a year ago while firing a yager, the but-end struck against the right collar bone, near its middle, followed instantly by great numbness of the whole limb, which continued upwards of an hour, when it gradually subsided. Early in April following, the arm began to swell from the wrist to the shoulder, attended with slight pain and impaired sensibility, which were supposed to be of a rheumatic character. The tumor was first perceived in June; it was situated just below the clavicle, pulsated very faintly, and did not exceed the volume of a hazel-nut. From this period on it gradually enlarged, having acquired by the following Christmas the dimensions of an apricot or moderate-sized plum. It had also become the seat of considerable pain; and there was a manifest increase of numbness in the affected side. In January the aneurism was observed to grow with great rapidity, the arm became gradually useless, and, in short, there was a decided aggravation of all the symptoms; the sufferings of the patient, when I first saw him at the date above specified, being of the most excruciating nature.

As there was considerable vascular excitement with a coated condition of the tongue and constipation of the bowels, he was requested to be bled immediately to the amount of about twenty ounces, to take a liberal dose of senna and salts, to keep perfectly quiet, and to use none but the lightest kind of food. On Wednesday the 17th of February he called again at my room, when it was found that the tumor had much increased in size, being nearly or quite one-third larger than on the previous Saturday. The pain was also much greater; his appetite was extinct; and for the last three or four nights he had scarcely slept a wink. Under these circumstances, convinced that no time was to be lost, I immediately requested a consultation with my colleagues, Professors Drake, Cobb, and Miller. Dr. Bayless, our Demonstrator of Anatomy, Dr. T. L. Caldwell, Dr. Donne, and several other physicians were likewise present, and kindly afforded their views concerning the case. After carefully deliberating upon the subject, these gentlemen unanimously concurred with me in the opinion that the only chance for the patient's life was to tie the subclavian artery; and the operation was accordingly appointed for twelve o'clock the next day.

On Thursday, February 18th., in the presence of my colleagues, a large number of physicians, and of the medical class, I proceeded to the operation. The patient was placed upon a narrow table of moderate height, the head and chest were properly elevated with pillows, and the face turned a little towards the sound side, while an assistant pulled at the wrist, so as to depress as much as possible the affected shoulder. The integuments over the clavicle being next stretched upon the chest, I made my first incision along the centre of that bone, beginning near the sternal origin of the mastoid muscle and passing out towards the acromion process of the scapula for about three inches and a half; thus dividing at one stroke the common integuments and the fibres of the platysma-myoid. The parts being now allowed to retract, left the lower margin of the cut parallel, and on a level with the superior border of the clavicle. A second incision, about two inches in length, was carried along the posterior edge of the mastoid muscle, at a right angle with the preceding. The triangular flap thus formed was then disssected up and held out of the way, care being taken not to interfere with the external jugular vein, or any of the smaller arteries of the neck. Having advanced thus far, the cervical aponeurosis was detached from the clavicle by cautious strokes of the handle of the scalpel, which laid bare the brachial plexus of nerves and the omo-hyoid muscle. At this stage of the operation a small vein, a branch of the subclavian, was divided, and although it bled very little, it was immediately secured with a temporary ligature. Taking the omo-hyoid for my guide, I divided the loose cellular substance in the triangular space bounded above by the muscle just mentioned, by the clavicle below, and by the anterior scalene muscle internally, and thus approached the artery as it passed over the first rib. The vessel here lay at some distance from the inferior branch of the brachial plexus of nerves, rather deeply behind the collar-bone; and with a common aneurismal needle, armed with a double ligature of saddler's silk, no difficulty was experienced in securing it, the instrument being passed from before backwards and from below upwards. It was then drawn very firmly with the fingers and tied with a double knot within a few lines of the anterior scalene muscle: as soon as this was accomplished all pulsation in the sac, as well as at the wrist, ceased. One end of the ligature being cut off, the other was left protruding at the inner angle of the wound, the edges of which were closed by three sutures and adhesive straps. Not half an ounce of blood was lost during the operation, which lasted twenty minutes.

The man was put to bed, and the limb, laid in an easy position, wrapped in cotton wadding. In less than an hour, the temperature, which had been considerably depressed, was thoroughly restored; the pain and numbness had greatly abated; and the patient expressed himself more comfortable than he had been for a month.

8 o'clock, P. M.—He is quite comfortable, except a slight degree of soreness along the wound. His breathing is natural, and the pulse about 80. He was requested to lie perfectly quiet, to keep the limb well covered, and to drink toast water.

19. 9 o'clock, A. M.—Had a pretty good night, having enjoyed several hours of sound and refreshing sleep. Tongue moist and tolerably clean: pulse 80: heat of the skin natural: no unusual thirst: temperature of the limb equal to that of the opposite side: *tumor quite solid.* Ordered thin gruel, as he expressed a wish to have something to eat.

8 o'clock, P. M.—Progresses very well; there being no fever, or any pain or tenderness in the wound: bowels have not been opened since the operation: is requested to take an ounce of sulphate of magnesia at 10 o'clock.

20. 9 o'clock, A. M.—Passed a good night: pulse soft, regular, and about 78 a minute: tongue moist and pretty clean: is free from pain, except at the back part of the shoulder and arm, where there is some degree of uneasiness: bowels relieved by the salts: numbness of the limb rapidly subsiding.

7 o'clock, P. M.—Has had another alvine evacuation: the wound exhibits a good appearance; and the patient is, in all respects, doing well.

21. 10 o'clock, A. M.—Removed the dressings, and cut away two of the sutures: the wound has united pretty well, except at each angle, where there is a discharge of healthy looking pus: parts beneath have a solid feel: tumor is considerably diminished in volume: patient rests well, and is entirely comfortable.

From this date every thing went on favorably. The tumor steadily decreased in size, the numbness of the limb grad ually subsided, the hand and fingers could be more easily moved, the temperature was equal to that of the opposite arm, the patient had a good appetite, and, in short, every function appeared to be well executed. He was still kept on the lightest kind of food, and was forbidden even to raise himself up in bed: no medicine was administered, excepting occasionally a dose of oil or salts, to regulate the bowels. The other stitch was removed in a few days, and the wound rapidly cicatrized, excepting at the upper angle, and at the point where the ligature of the artery protruded.

The ligature came away on the morning of the fourteenth day from the operation, without a drop of blood. The patient at the time was in good health and spirits, and expressed a wish to return to his family, upwards of one hundred miles down the Ohio. The tumor had diminished fully one-half in volume, the pectoral muscle, beneath which it was situated, being quite flaccid and extensible. The pulse at the wrist was still absent, and there was likewise some numbness at the top of the shoulder and the extremities of the fingers, but ihe movements of the limb daily improved, and the patient was perfectly convalescent. To guard against accident, he was requested to live low, and keep his bed for a few days longer.

On Sunday the 7th of March, being obliged to leave town, I placed the patient in the care of my friend Dr. T. L. Caldwell, to whom, as well as to Dr. S. B. Richardson, who was subsequently called in, I am greatly indebted both for his unwearied attention and skilful treatment, and for an account of the daily progress of the case until my return. From the diary furnished by the former gentleman I make the following extracts.

Nothing worthy of notice occurred until Saturday night the 13th of March, when he began to feel somewhat unwell; he had walked about the room occasionally, and been out several times in the yard. Being no better next day, he took a dose of castor-oil of his own accord, and on Monday morning an ounce of salts, both of which had operated well, and he expressed himself much relieved. He had experienced no pain, and attributed his indisposition to having taken cold from throwing off the bed-clothes on Sunday night. Dr. Caldwell, who saw him towards noon on Monday, found his pulse somewhat accelerated, with a slight degree of tenderness in the apex of the tumor, the volume of which had not perceptibly diminished since my departure. The wound was perfectly cicatrized, excepting at two points not exceeding a quarter of an inch in length, and from which there was a very small discharge of healthy matter. The pulse at the wrist was still imperceptible.

16. 5 o'clock, A. M.—About 12 o'clock last night the patient was suddenly seized with intense pain in the chest, which was particularly severe at the base of the right lung, from which it extended over towards the sternum, on the one hand, and up towards the axilla, on the other. Respiration short, hurried, laborious, and about 56 a minute: pulse 140, quick and tense: tongue moist and covered with a white coat: aneurismal tumor had suddenly disappeared last night at the time of the attack. Dr. Richardson having reached the patient a short time before Dr. Caldwell, they adopted the following treatment: bleeding at the left arm to about sixteen ounces, and the subjoined prescription, which was ordered to be repeated every two hours:

℞ Subm. Hydrarg. gr. iv.
Nitr. Potassæ gr. iii.
Antim. Tartariz. gr. ¼. M.

In addition to this, a solution of antimony, in the proportion of one grain to the ounce of water, was ordered to be given, in teaspoonful doses, at the alternate hour, to keep up slight nausea. The bleeding produced considerable relief of the pain, with a diminution of the tenseness and frequency of the pulse.

3 o'clock, P. M.—The pain, though somewhat abated, still persisting, he was cupped to six ounces at the lower part of the chest with a good deal of relief: had a small alvine dejection in the forenoon: suspend the mercurial powders, and continue the antimonial mixture.

6 o'clock, P. M.—Bronchial respiration throughout the right lung, with dulness on percussion over the lower ribs: the blood drawn this morning sizy and cupped. Ordered a large blister to the side, extending from the base of the chest to a short distance above the nipple: continue the antimony every hour.

17. 8 o'clock, A. M.—Blister has drawn well: there is some abatement of pain, the respiration is less frequent, and the pulse is also somewhat better: continue the antimonial mixture steadily, but give it in larger doses to produce nausea.

6 o'clock, P. M.—There is more vascular excitement: no motion from bowels since yesterday: twelve ounces of blood were taken from the arm, the pulse giving way under it: persevere with the previous medicine, and administer half an ounce of castor-oil, repeating it every three hours, if necessary.

18. 8 o'clock, A. M.—Blood drawn last evening buffed: bowels not yet moved: the patient experiences a sensation near the superior part of the chest as if a fluid was passing from the pleuritic cavity into that of the aneurismal tumor, and on carefully auscultating the spot, Dr. Caldwell could distinctly hear a plashing sound at every inspiration, the noise resembling that produced by shaking water in a closed vessel: the respiration is still bronchial, though not so hurried and painful, and the dulness on percussion is perceived over a larger extent of surface than previously: the breathing is likewise of a bronchial character in the left lung: ordered one ounce of sulphate of magnesia, another blister to the upper part of the chest, and a continuance of the antimony.

6 o'clock, P. M.—Medicine has operated two or three times on the bowels: blister has drawn well: tongue coated and rather dry: *no pain in the chest:* breathing slightly improved.

19. 8 o'clock, A. M.—Had a tolerably good night: respiration still further improved: tongue dry throughout: pulse 140: continue the antimony.

2 o'clock, P. M.—Having returned to the city, I visited Daniel with Dr. Caldwell, Dr. Richardson, and Prof. Miller: pulse small and 140: breathing short, laborious, and 38 in a minute: extremities cold: surface bedewed with clammy perspiration; tongue dry and covered with a brownish-white coat: bronchial respiration in left lung, and almost complete absence of sound in the right: ordered

℞. Carb. Ammoniæ ʒ ii.

Pulv. G. Acaciæ ʒ i.

Mist. Camph. ℥ vi. M.

Give a large table spoonful every hour, and drink milk punch freely.

9 o'clock, P. M.—Condition unchanged: continue as before.

20. 9 o'clock, A. M.—Appears to be somewhat better, the pulse having more volume and less frequency: dozed some during the night: has had five or six alvine evacuations since last evening: is very feeble, but still sensible: continue with the ammonia and milk punch, and take ten drops of laudanum every two hours until the diarrhœa is checked.

2 o'clock, P. M.—Continues much the same, excepting that he is sensibly weaker. Towards evening the mind began to wander, deglutition became difficult, the pulse ceased in the left wrist, and about nine o'clock in the evening he expired apparently without pain; having survived the operation a little upwards of thirty days.

It is worthy of remark that the limb of the affected side retained its temperature fully up to the last moment, although the pulse at the wrist was at no time perceptible. It should also be observed that there was scarcely any cough, or expectoration, during the whole of the period which intervened between the rupture of the aneurismal sac and the patient's death.

*Autopsy.*—At my particular request the body was inspected, thirty-six hours after death, by Dr. T. L. Caldwell, Dr. Richardson, and Dr. Bayless, in the presence of a large number of the most respectable practitioners of the city, and several medical students. The preceding evening a cold injection of lead had been thrown into the innominata with such force as to pass into the brain and return by the left carotid and vertebral, at the same time filling pretty completely the arteries of the neck, the shoulder, and superior extremity of the affected side.

The body was somewhat emaciated; the wound made during the operation completely cicatrized; and the pectoral muscles considerably wasted, but in other respects unchanged. The subclavian artery terminated abruptly at the outer margin of the scalene muscle, where the ligature had been applied, *its caliber being closed by a mass of solid fibrin, about one-third of an inch in length, which adhered firmly to the lining membrane, and thus afforded an effectual barrier to the passage of the blood.* Between this and the thyroid axis the vessel was occupied by a dark coagulum of blood, which, as it was unadherent, was probably formed only a short time before death. Beyond the seat of the ligature the artery had a rough, ragged appearance, and was sufficiently pervious to admit of the ready passage of a small probe into the aneurismal sac. Superiorly the tumor was overlapped by the brachial plexus of nerves, while in front, at its lower part, was the subclavian vein, which, besides being thrown out of its natural course, was considerably diminished in size. No pus was any where discoverable, the parts immediately involved in the operation being intimately consolidated by plastic lymph. The aneurismal tumor, placed immediately below the clavicle, was of a conical form, and about the volume of a moderate-sized orange, being two inches and a quarter in diameter at its base. Its walls varied in thickness at different points from half a line to the eighth of an inch, and its interior communicated by means of oval aperture, one inch and three quarters in length by an inch and a half in width, with the pleuritic cavity: it was situated between the first and second ribs, nearly equi-distant between the sternum and the spine, and was the result obviously of ulcerative absorption induced by the pressure of the tumor. Both ribs were denuded of their periosteum immediately around the opening, and the serous membrane had a shreddy, ragged aspect. The aneurismal sac contained a few reddish clots arranged in a laminated manner, and closely adherent to its inner surface, especially at the part corresponding with the apex of the tumor.

The right thoracic cavity contained nearly three quarts of bloody looking serum, intermixed with flakes of lymph and laminated clots, the latter of which were of a reddish-brown color, and had evidently been lodged originally in the aneurismal sac. The pleura exhibited every where marks of high inflammation, and the right lung was greatly reduced in volume, from the compression of the effused fluid. The left lung was considerably engorged, and at one or two points almost hepatized. The heart and pericardium were sound. The abdominal viscera presented nothing unusual. None of the arteries appeared to have been affected by disease.

The vessels which, by their communication with the subclavian artery above the ligature, and the axillary trunk below the tumor, were mainly instrumental in keeping up the circulation of the limb and shoulder, were the supra and infra scapular, together with the short thoracic. The former of these branches arose on the tracheal side of the scalene muscle, and passing over its anterior surface, about three quarters of an inch above the clavicle, soon separated into three twigs, one of which ran towards the root of the coracoid process, the other two towards the posterior part of the scapula. The truuk of the vessel did not appear to be particularly enlarged, but its terminal divisions were nearly twice the ordinary volume. The infra-scapular artery, which arose from, and consequently opened into, the lower part of the sac, was nearly as capacious as the axillary, or parent trunk. It soon divided into two large branches, one of which formed the long thoracic artery, while the other was destributed to the subscapular muscle. The short thoracic vessels presented nothing worthy of note.

In reflecting upon the manner in which this case terminated, the question naturally arises, did the ulcerative absorption which gave rise to the opening above referred to, and which finally led to the escape of a portion of the contents of the aneurismal sac, commence prior to the deligation of the subclavian artery on the 18th of February, or not until sometime subsequently? For my own part, I am disposed to believe that it was set up fairly before the operation was performed; a view of the case which is fully borne out, I think, by several circumstances, such especially as the extraordinary bulk of the tumor, and the violent pulsation that was constantly going on in its interior. Under the influence of these two causes, ulcerative absorption must have commenced, in all probability, several months—certainly six or seven weeks—before the occurrence of the perforation which ultimately destroyed the patient. As soon as the operation was over all pulsation in the tumor ceased; its contents became speedily solidified; and its volume progressively diminished, so that for upwards of three weeks there was every reason to believe the patient would rapidly recover. Indeed, no case could have presented more flattering prospects. After the artery was tied the pressure occasioned by the swelling must have been comparatively slight, as was evinced by the fact that the pain and numbness, which before had been constant and excessive, almost immediately subsided. Hence it follows that ulcerative absorption would be much more likely to have been induced before than after the operation. The denuded condition of the ribs, the ragged state of the neighboring pleura, and the absence of purulent matter in the sac and in the wound, the latter of which was perfectly healed when the man died, all concur, moreover, to induce the belief that the perforating process had been going on for sometime prior to the performance of the operation.

Another question of no little interest in reference to the case before us, may be asked here, namely, what would have been the probable effect of opening the aneurismal sac and clearing it of coagulated blood, soon after the artery was tied? Could the event which occurred have been foreseen, such a procedure, I conceive, would not only have been warrantable, but highly proper; but under the circumstances it would not have been right, certainly not in accordance with the general experience of the profession on this point, to attempt it. The practice, once so much in vogue, of laying open the sac must, to say the least of it, be a very dangerous one; that it was almost universally so previously to the time of John Hunter is too well known to require any comment on this occasion, and that it would be less so at the present day, even with our improved methods of operating, admits, I presume, of much doubt.

Be this as it may, the case, considered in relation to the manner in which it terminated, may be regarded as altogether unique. The only instance, so far as my information extends, which is at all similar to it is one narrated by Mr. Guthrie, in his work on the diseases and injuries of the arteries. As it is highly important both in a pathological and practical point of view, no apology need be offered for introducing a brief outline of it in this place.

A stout, middle-sized man, 53 years of age, became a patient in one of the London hospitals, on account of an aneurismal swelling immediately below the right clavicle, which was first noticed in November, soon after lifting a heavy weight. It gradually increased in size until the following August, when, unwilling to submit to an operation, he went into the country. In December he returned in great suffering, and requested the assistance of Mr. Guthrie. He now experienced continued pain and numbness in the shoulder, chest, and arm of the affected side, with a teazing cough and purulent expectoration; the tumor, which pulsated strongly and with a peculiar thrill, was of the volume of a forty-eight pound shot; and the clavicle was pushed high up into the neck. In a few days after this the patient began to spit blood, which gradually increased in quantity until his death, which happened in three weeks from the time of his return to London. On opening the body it was found that the aneurism had forced its way into the right thoracic cavity by the removal of a part of the first five ribs, had contracted adhesions to the superior lobe of the lung, and had finally opened into it, so that the gradual discharge of blood destroyed the patient.

### II.—*Cases of Axillary Aneurism for which the Subclavian Artery was Tied by other Surgeons.*

I shall now proceed to present an account of some of the cases in which this operation has been performed by other surgeons. A summary is of course all that can be expected, my design being merely to point out the manner in which these attempts have terminated, and to exhibit, as far as this can be done from the imperfect data before me, some of the most important facts in reference to the history of axillary aneurism. After ransacking all the surgical works and medical periodicals within my reach, and these are not few in number, twenty-seven cases are all I can find, and the greater part of these, unfortunately, are devoid of the proper details.

*Case* 1.—The first case in which an attempt to tie the subclavian artery, by cutting above the clavicle, was accomplished, is that of Mr. Ramsden, of London.* John Townley, a tailor, aged thirty-two, of very intemperate habits, and peculiarly unhealthy aspect, was admitted into St. Bartholomew's Hospital, Tuesday, November 2, 1809, on account of aneurism in the right axilla. The most prominent part of the tumor was about half as big as a large orange, and there was

*Ramsden's Practical Observations on Surgery, p. 276.

also much distension underneath the pectoral muscle, so that it was impossible to bring the elbow near the side of the body. The limb, although it retained its temperature, soon became œdematous, and the pulsation of the radial artery, already very faint, at length entirely ceased. On the evening of the twelfth day a dark spot appeared on the centre of the tumor, surrounded by inflammation, which threatened more extensive destruction of the skin. A farther postponement of the operation being deemed inadmissible, Mr. Ramsden performed it the next day, tying the artery on the outside of the scalene muscle. The shoulder being considerably elevated by the tumor, great difficulty was experienced in passing the ligature; this, however, was finally overcome by a probe of ductile metal and a pair of small forceps. As soon as the artery was tied, the pulsation in the swelling ceased; the limb afterwards fully retained its temperature; and the tumor had already somewhat diminished in volume, when about five days after the operation the patient expired. On examination, it was ascertained that the subclavian artery, where the ligature was applied, was so nearly separated that it was held together only by a few shreds of dead matter. Each extremity of the vessel appeared to be completely consolidated and impervious, leaving no doubt of its being fully competent to resist the impetus of the blood from the heart. The aneurismal sac contained about two pints of blood, the greater part of it in a fluid state; the heart and large vessels were perfectly sound, even the subclavian, excepting at the side of the ligature; and the collateral vessels did not seem to have acquired any increase of capacity beyond what is natural to them.

*Case* 2.—In the year 1811, the subclavian artery was tied in the London Hospital, in a case of axillary aneurism, by Sir William Blizard. The patient, who was old and debilitated, died on the fourth day, though strong hopes were at first entertained of recovery.*

*Case* 3.—A clergyman, 48 years of age, of a stout athletic frame, and who had always enjoyed the most robust health, was affected with axillary aneurism, in consequence of a fall from his horse in December, 1812. In July following the tumor was fully as large as a goose egg: on the sixteenth of that month Dr. Colles, of Dublin, secured the right subclavian artery with two ligatures on the acromial side of the scalene muscle. The time occupied by the operation is not stated. Death occurred on the fourth day from inflammation of the chest and limb.†

*Case* 4.—In 1815 Mr. Thomas Blizard secured the subclavian artery in the London Hospital. The case was one of aneurism in the left axilla; the ligature was applied at the usual point "with great facility:" and the patient died on the evening of the eighth day, previously to which event the ring and middle fingers turned black. On dissection the pericardium exhibited the effects of high inflammation; the heart, of a deep red color on its posterior surface, was covered with flakes of lymph; the lining membrane of the aorta, right carotid and left subclavian was of a scarlet complexion, and the boundaries of the aneurismal tumor were in a state of sphacelation.‡

*Case* 5.—This was the first case in which complete success attended the deligation of the subclavian artery, immediately after its exit from between the scalene muscles. The patient was a gentleman, with an aneurism in the left axilla. The tumor was of large size, and had encroached so much upon the clavicle as to throw it considerably above its natural level.

*Hodgson on the Diseases of the Arteries, &c.
†Edinb. Med. and Surg. Journal, vol. 2, p. 14.
‡Hodgson, op. cit. p. 602.

The ligature was passed under the artery with great facility, and immediately on tying it all pulsation in the brachial and radial arteries ceased. In the afternoon the temperature of the limb was observed to be rather higher than that of the other arm. The operation was performed by the late Professor Post, of New York, on the 8th of September, 1817. On the 17th the aneurismal tumor burst, followed by a discharge of about three ounces of dark grumous blood. On the 26th the ligature was detached from the subclavian artery; and before the middle of October the wound was perfectly cicatrized.*

*Case* 6.—On the 7th of March, 1819, Baron Dupuytren, surgeon to the Hotel-Dieu of Paris, tied the subclavian on the left side, just as it emerges from behind the scalene muscle, in a case of false aneurism of the axillary artery. The patient was of an excellent and robust constitution, thirty-seven years of age. Having been a soldier, he received, seven years ago, in a charge of cavalry, a thrust from a sword which penetrated the axillary artery. The tumor was about the size of a child's head at the time of the operation, which lasted one hour and a quarter. About the fifteenth day the ligature came away; and on the 27th of March the wound was nearly healed. The patient made a perfect recovery.†

*Case* 7.—On the 30th of March, 1819, Dupuytren performed his second operation for axillary aneurism. The patient, formerly a soldier, forty years of age, and laboring under constitutional syphilis, received a wound from a flat-bladed lance in 1815. The tumor remained small for a considerable period, and then suddenly attained a great volume, extending itself under the pectoral muscle, and elevating the clavicle.

*Cooper's Surgical Dictionary.
†Edinb. Med. and Surg. Journal: vol. 15, p. 476.

The ligature was applied on the acromial margin of the scalene muscle, after one hour and forty-eight minutes; and the case terminated fatally on the ninth day, the swelling having been invaded by gangrene, and the patient having experienced several severe attacks of hemorrhage from the wound.*

*Case* 8.—The next case is that of Robert Liston, Esq., of Edinburgh, who tied the subclavian artery on the third of April, 1820. This was the first successful operation of the kind in Scotland. The patient, Alexander Gibson, a coachman, aged 35 years, referred the origin of his disease to a fall five months ago in which he pitched on the left shoulder. Latterly the swelling increased with great rapidity, and seemed to extend above the clavicle, which it had very considerably displaced upwards. During the operation, which lasted altogether about half an hour, the anterior scalene muscle was divided. The ligature separated on the twelfth day, and the wound was completely cicatrized by the 8th of May, the tumor having in the meanwhile greatly diminished in size.†

*Case* 9.—John Robertson, a porter, aged 47, was admitted into the Royal Infirmary of Edinburgh, on the 7th August, 1823, for an aneurism of the left side, extending along the lower margin of the clavicle to the axilla. The tumor arose without any assignable cause, and when first noticed, about three weeks before, was scarcely larger than a bean. On the 23rd, Dr. Wishart secured the subclavian artery external to the scalene muscle, the operation occupying only "a short time." On the sixteenth day after the ligature was found lying loose in the wound; and a rapid recovery followed.‡

*Case* 10.—In a patient upwards of sixty years of age, in St. Thomas' Hospital, Mr. Travers tied the subclavian artery,

---

*Edinb. Med. and Surg. Journal, vol. xvi, p. 199.

†Op. cit. vol. xvi, p. 348.

‡Op. cit. vol. xxi, p. 15.

January 17th, 1823, for axillary aneurism. The great elevation of the clavicle rendered the operation very difficult, independently of the profuse hemorrhage. Altogether it occupied upwards of two hours. The patient died on the third day.*

*Case* 11.—George Vaughan, aged 36, a man of robust frame, an extra-tide waiter by occupation, lacerated his axillary artery in July, 1823, while making a sudden exertion with his arm. An aneurismal tumor rapidly formed, which by the end of August was about four inches in diameter, distinctly circumscribed, and extending from the lower edge of the clavicle to the lower border of the pectoral muscle. On the 20th of September, Charles Aston Key, Esq. of London, tied the right subclavian on the acromial side of the scalene muscle, the operation lasting twenty minutes. The ligature was detached on the twelfth day, and the patient speedily recovered. This appears to be the first case in which this vessel was secured with success in the London hospitals.†

*Case* 12.—Thomas Overall, brass-founder, aged 33, was admitted into St. Thomas' Hospital, August 1st, 1825, under the care of Mr. Green, for aneurism of the right axillary artery. The tumor had existed upwards of five months, and was about the size of a large orange. His shoulder had been dislocated sometime previously, and to that accident he attributed his present disease. "A long time elapsed in coming down to the artery," which was finally secured at the usual situation. Every thing went on well until the thirteenth day, when secondary hemorrhage set in, which considerably weakened the patient, and rendered the success of the operation for a while doubtful. On the eighteenth day the wound was nearly healed, but the ligature had not yet separated from the artery.‡

*London Medico-Chir. Mag.—Philad. Jour. Med., vol. vii, p. 183.

†Trans. of the Royal Med. and Chirurg. Society, vol. xiii.

‡Lancet, vol. viii, pp. 189–283.

*Case* 13.—John McIntyre, aged 43, presented himself to Mr. Liston, of Edinburgh, September, 1826, with a very large aneurismal tumor in the right axilla, equalling in volume a common-sized foot-ball. It passed upwards underneath the collar-bone, which it had raised considerably, and downwards below the border of the axilla, pressing in the ribs and flattening the chest. About Christmas, 1825, he fell on the ice with his arm stretched out, and to this accident he ascribed his disease. On the 12th September Mr. Liston tied the subclavian artery at two points, the last close to the outer edge of the scalene muscle; and, although every thing promised well for a while, yet death occurred on the fourteenth day, preceded by slight hemorrhage from the wound. On dissection the vessel was found to be considerably diseased; the wound was filled with coagula; and at neither of the points of deligation was there much appearance of reparation.*

*Case* 14.—Thomas Wharnby, aged 36, was admitted into the Manchester Infirmary, April 16, 1827, in consequence of an aneurismal tumor in the right axilla as large as a moderate-sized orange. With the exception of this disease, which was first noticed about twelve months ago, he was apparently healthy. On the 17th of June Robert Thorpe, Esq. tied the subclavian artery at the place usually selected for that purpose, the whole operation occupying fifteen minutes. The ligature separated on the 4th of July, and the patient got entirely well.†

*Case* 15.—William Weston, wood-hawker, aged 38, a stout and very muscular man, was admitted into Guy's Hospital in November, 1827. The aneurism, which made its appearance

*Edinb. Med. and Surg. Journal, vol. xvii, p. 4.
†Medico-Chir. Review, January, 1828.

without any assignable cause about nine weeks previously, occupied the right axilla, and was about the size of an egg. The operation of tying the subclavian artery was performed, in the usual manner, by Bransby B. Cooper, Esq. on the fourth of December, and the patient had a speedy recovery.*

*Case* 16.—Professor Gibson, of the University of Pennsylvania, tied the subclavian artery on the 17th of March, 1828, for axillary aneurism in John Langton, a muscular athletic man, a laborer by occupation, aged thirty-five. The disease was produced two days previously in an attempt to reduce an old luxation of the left shoulder-joint. The vessel was secured close to the outer margin of the scalene muscle; and the patient died on the 23d of March, the eighth day after the operation. The left hand and fore-arm were in a state of incipient gangrene, and no coagulum or trace of lymph could be observed above the ligature.†

*Case* 17.—Hermenegildo Gonzales, a poor farmer, the father of three children, of small stature and spare habit, sixty-one years old, had always enjoyed good health until October 1827, when he began to feel pain along the course of the scalene muscles of the right side, which he supposed might have been occasioned by violent horse-back exercise. This was soon followed by the developement of a tumor in the axilla, which by the early part of the following April was of a hemispherical form and measured three inches in diameter. The subclavian artery was tied by Dr. Edward W. Wells, of Maracaybo, on the 12th of April, on the acromial side of the scalene muscle; the ligature came away on the third of June, by which time the tumor had decreased to the size of a walnut; the wound was perfectly cicatrized by the fourteenth of that

*London Medical and Physical Journal, February, 1828.
†American Journal of the Medical Sciences, vol. ii, p 136.

month; and the patient lived nearly three years after the operation, dying finally of ulceration of the bladder.*

*Case* 18.—A stout muscular individual, a fisherman by occupation, aged forty-six, was admitted into the Parochial Infirmary at Davenport, England, June 10th, 1830, with a large, tense, pulsating tumor on the right side, extending from the cartilage of the fifth rib to the axilla over the point of the shoulder. It made its appearance about thirteen weeks previously, soon after an attack of rheumatism of the right arm; in a month it was of the size of a walnut, and in a fortnight thereafter it attained its greatest volume. On the 23d of June, Mr. Crossing tied the subclavian near its exit from between the scalene muscles, the whole operation lasting twenty-five minutes. The ligature came away on the *eighty-fifth* day, and in December following the tumor had nearly disappeared.†

*Case* 19.—William Hines, of Smithville, Virginia, laborer, twenty-eight years of age, became a patient of Professor Mott of New York, August 24th, 1830, for axillary aneurism of the right side. The tumor was about the volume of a goose-egg, and made its appearance seven weeks ago, soon after a violent strain produced by carrying a canoe on hand-bars across the arms. His general health was good. The artery was tied a little external to the scalene muscle, and at the expiration of twenty-seven days the wound was perfectly healed; the aneurismal tumor being very hard and much diminished in volume. The operation lasted fifteen minutes.‡

*Case* 20.—The subject of this case was a man sixty-three years of age, who attributed his disease to a hurt received

*Amer. Jour. Med. Sciences, vol. iii p. 28. See also vol. xiii, p. 406.
†Medico Chirurg. Review, vol. xv, p. 507.
‡American Jour. Med. Sciences, vol vii, p. 309.

sometime before. The tumor, which resembled the section of an oblate spheroid, extended from the clavicle into the axilla of the right side, being about the size of a large orange. The operation of tying the subclavian artery was performed on the 17th of December, 1830, by W. Bland, Esq. of Australia; and the ligature fell off on the 29th of January, leaving the wound entirely healed.*

*Case* 21.—In 1830 William H. Porter, Esq., of Dublin, tied the left subclavian successfully at the Meath Hospital, in a man forty years of age. The aneurismal tumor was situated at the part corresponding to the division between the great pectoral and deltoid muscles, had a firm pulsating feel, and was nearly of the volume of a goose-egg, having slightly displaced the clavicle. The ligature was applied at the usual point, and for about a month every thing progressed favorably. At the end of this time the tumor, which had diminished in size, began to increase and become painful, and, notwithstanding the diligent employment of antiphlogistic measures, went on to suppuration. An incision being made into it, it discharged about a pint of fœtid purulent matter, mixed with large dark clots of blood. Under the use of pressure it soon healed, and the man eventually recovered.†

*Case* 22.—In this case the artery was secured by Charles Mayo, Esq. of Winchester, England, March 26, 1831. The patient, W. Mathews, was forty-nine years of age, and the tumor, which was of about six weeks' standing, occupied the left claviculo-axillary region. The ligation was completed in a little more than twenty minutes. On the 10th of May the

*American Jour. Med. Sciences, vol. ix, p. 527. See London Med. and Surg. Journal, Oct. 1831.

†Dublin Hospital Reports, vol. v, 1830. North American Med. and Surg. Journal, vol. xii, p. 106.

wound was perfectly healed, and the tumor had disappeared.* Mr. Mayo operated, but unsuccessfully, on a similar case ten years before, the particulars of which I have not been able to obtain.

*Case* 23.—The next case of axillary aneurism in which the subclavian artery was tied, is that of William Ferguson, Esq. of Edinburgh. The patient, Mark Robson, a waiter, aged sixty, of intemperate habits, could assign no cause for the original appearance of the disease, but thought it might have resulted from some injury on the arm during one of his drunken fits. The tumor, which was scarcely perceptible for many months, rapidly increased within the last six weeks, and was now about the size of a large fist. The operation was performed in the usual manner on the 12th of May, 1831; the ligature separated on the 13th of June; and a rapid recovery followed.†

*Case* 24.—Robert Blackwood, a weaver, aged sixty-five, was admitted into the Royal Infirmary of Glasgow in June, 1833, on account of a strongly pulsating tumor under the left clavicle, extending towards the lower part of the axilla. It was of an oblong, pyriform shape, measuring fully six inches in one direction by two and a half in the other. The swelling first appeared *above* the clavicle eighteen months ago, and, with the exception of cough and dyspnœa, both of which were aggravated on assuming the horizontal posture, the health of the patient was quite good. Dr. William Auchincloss tied the subclavian artery *within* the outer-third of the anterior scalene muscle, the operation, dressing and all, occupying about forty minutes. Death occurred in sixty-eight hours and a half, from effusion of serum on the surface and into the ventricles of the brain.‡

*Medico-Chir. Review, vol. xv, p. 482.
†Edinb. Med. and Surg. Journal, vol. xxxvi, p. 309.
‡Edinb. Med. and Surg. Journal, vol. xlv, p. 324.

*Case* 25.—Mrs. Hain, a weaver, of a spare habit of body, aged forty-two, had a pulsating tumor in the left axilla, which, on the 16th of April, 1834, was about the size of a man's fist. She could assign no cause for its origin, and first perceived it about ten years ago, when it was very small, and produced little or no inconvenience, even when engaged at her usual employment. Within the last six months it has increased with considerable rapidity, and eight days ago, when she experienced a sensation as if something had given way, she observed that the swelling had assumed a different shape and was much larger than previously. The local suffering also greatly augmented, and the general health became sensibly impaired. The subclavian artery was tied by Professor Lizars, of Edinburgh, on the 27th of April, on the acromial side of the anterior scalene muscle, in the short space of ten minutes. Scarcely any blood was lost; about sixty hours after the operation the pulsation could be felt in the arteries at the wrist; and the woman rapidly recovered without any untoward occurrence.*

*Case* 26.—The next case in which the subclavian was tied, and the last which I shall mention on this occasion, is that narrated by Dr. Samuel Hobart, of Cork. The tumor, which was situated immediately under the right clavicle, near its acromial extremity, was of the volume of a large duck-egg, and was first observed about four months previously, without any assignable cause. The patient, John Wright, aged 38, was "a respectable citizen of Cork, and of good bodily habits." The artery was readily secured on the acromial side of the scalene muscle on the 7th of May; the ligature was detached about the sixteenth day; and on the 30th of July the tumor had almost wholly disappeared.†

*Lancet, 1834, vol, ii, p. 717.

†Edinb. Med. and Surg. Journal, vol. xlv, p. 48.

From the preceding abstract it will appear that there was only one female in the twenty-six cases. Axillary aneurism may therefore be regarded as being much more common in men than in women; agreeing, in this respect, with aneurism of the carotid, iliac, femoral, and popliteal arteries, as well as with that of the aorta.

Of these twenty-six patients, seventeen, or nearly two-thirds, were *cured*. Of the remaining nine, two died on the third day; one on the fifth; two on the fourth; two on the eighth; one on the ninth; and one on the fourteenth. The cause of death, as might be expected, was not alike in all the cases. In one—that of Dr. Auchincloss—it evidently resulted from the effusion of serum on the surface and into the cavities of the brain. In Professor Gibson's case, the left hand and fore-arm were in a state of incipient gangrene, and no effort had been made at reparation, although the patient lived until the eighth day. In John McIntyre, on whom Mr. Liston operated in 1826, slight hemorrhage occurred a short time before death, which happened on the fourteenth day; and on dissection it was found that the artery had given way in consequence of ulcerative absorption. In this case, too, scarcely any attempt had been made at restoration. In case vii, death occurred on the ninth day, preceded by several slight attacks of hemorrhage from the wound, and by gangrene of the aneurismal tumor. In the patient operated on by Dr. Colles, violent inflammation was discovered in the chest and limb; and in the case of Mr. Thomas Blizard, there were evidences of severe pericarditis and arteritis, with sphacelation of the aneurismal swelling and of the ring and middle fingers. In the other two cases no mention is made of the cause of death.

In ten of the cases, the disease occurred on the *left side*, in twelve on the right. The *youngest* of the patients was twenty-eight years of age; nine were between thirty and forty; eight between forty and fifty; and five between sixty and seventy; the oldest being sixty-five. In regard to their *occupation*, two were soldiers, one a gentleman, one a tailor, one a clergyman, one a coachman, one a farmer, one a wood-hawker, one a fisherman, one a tide-waiter, one a brass-founder, two weavers, two laborers, and two porters.

The *cause* of the disease is distinctly stated only in ten of the cases. In most it was produced by external violence, as a fall or strain. In the case of Professor Gibson, the tumor was developed soon after a fruitless attempt at reducing an old dislocation of the shoulder-joint; and in the soldiers operated on by Dupuytren the lesion was of a traumatic kind, the axillary artery being perforated by a sharp-pointed instrument. In one of the cases, that of a fisherman, forty-six years of age, the disease was attributed to the effects of rheumatism.

The *duration* of the disease varied from a few days to several years. In the case reported by Dr. Gibson, already alluded to, it was only of two days' standing; while in one of Dupuytren's it had existed for seven years, and for ten years in the one which fell under the observation of Professor Lizars. In this case, No. xiv, the aneurism came on without any assignable cause, and remained small until within six months of the time of the operation. In the other fourteen cases, in which I have been able to determine this point, the duration of the disease, prior to the deligation of the subclavian artery, was as follows:

| | | |
|---|---|---|
| In | 1 - - - - | 5 months. |
| " | 1 - - - - - | 5 weeks. |
| " | 1 - - - - | 2 months. |
| " | 1 - - - - - | 8½ months. |

| | | |
|---|---|---|
| In 1 | - - - - - | 12 months. |
| " 1 | - - - - - | 10 weeks. |
| " 1 | - - - - - | 7 months. |
| " 1 | - - - - - | 7 weeks. |
| " 1 | - - - - - | 3 months. |
| " 1 | - - - - - | 6 weeks. |
| " 1 | - - - - - | 18 months |
| " 1 | - - - - - | 4 months. |
| " 1 | - - - - - | 4 years. |

With respect to the *volume* of the aneurismal tumor, no definite information can be derived from the above cases, owing to the vague and unsatisfactory manner in which this subject has been treated by the reporters. Of the twenty-one cases in which this point is noticed, the following table may be presented, it being impossible to deduce any general conclusion from them.

| | | | |
|---|---|---|---|
| In case | 1 | the tumor was | "large." |
| " " | 3 | " " | of the size of a goose-egg. |
| " " | 5 | " " | "large." |
| " " | 6 | " " | child's head. |
| " " | 7 | " " | "very large." |
| " " | 8 | " " | "large." |
| " " | 10 | " " | "large." |
| " " | 11 | " " | four inches in diameter. |
| " " | 12 | " " | very large. |
| " " | 13 | " " | foot-ball. |
| " " | 14 | " " | orange. |
| " " | 15 | " " | egg. |
| " " | 17 | " " | three inches in diameter. |
| " " | 18 | " " | "large." |
| " " | 19 | " " | goose-egg. |
| " " | 20 | " " | large orange. |

| | | | | | |
|---|---|---|---|---|---|
| In case | 21 | the tumor | was | of the size of a | goose-egg. |
| " " | 23 | " | " | - - | large fist. |
| " " | 24 | " | " | | six inches by two and a half. |
| " " | 25 | " | " | - - - | fist. |
| " " | 26 | " | " | - - - | duck-egg. |

The length of time occupied in *performing the operation* is stated only in eleven cases, in which it varied from ten minutes, the minimum, to upwards of two hours, the maximum; the operator, in the former case, being Professor Lizars of Edinburgh, in the latter, Mr. Travers of London. Baron Dupuytren also consumed one hour and forty-eight minutes in one of his operations. In the remaining eight cases, the deligation of the subclavian occupied, in two, twenty minutes; in two, fifteen minutes; in one, twenty-five minutes; in one, forty minutes; and in two, thirty minutes.

In twelve of the cases the *ligature separated*, in three, on the twelfth day; in one, on the fifteenth; in two, on the sixteenth; in two, on the eighteenth; in one, on the thirty-second; in one, on the forty-third; and in one—that of Mr. Crosssing—on the eighty-fifth.

Of the *symptoms* of this lesion very little need be said in this place, as they are usually so well marked as to render it impossible to mistake them. When arising spontaneously, or without any assignable cause, it may exist for several months without attracting any particular notice, and the same thing occasionally occurs when it results from external violence, as a blow or strain. Generally, however, the tumor rapidly aug ments in bulk, and produces such a train of phenomena as to lead at once to its detection. Of these, one of the earliest, and at the same time most unpleasant, is the sense of fatigue or uneasiness in the affected part arising from the pressure on the axillary plexus of nerves. This symptom is usually succeeded in a few weeks, sometimes indeed in a few days, by a feeling of pain, which is always in direct ratio to the size of the aneurism, being comparatively slight when it is small, and more or less intense when large. Nor is the pain confined to the site of the disease; in most cases it radiates from it, as from a centre, in different directions, outwards into the shoulder, downwards along the arm, and upwards into the neck. Pressure, severe coughing, the recumbent posture, and the weight of the limb greatly increase it. It is also apt to be worse at night, and in damp states of the atmosphere. Numbness of the shoulder, chest, and arm, is another symptom which generally manifests itself at an early period of the disease, and is never absent when the tumor has acquired considerable magnitude. It is always very distressing to the patient, is greatly aggravated by pressure on the swelling, and commonly extends to the ends of the fingers. Indeed it is at this point that the sensation in question is often most keenly felt.

The pulsation of the tumor, at first faint and scarcely perceptible, becomes very distinct in the progress of the disease, so that it can be felt not only by the fingers, but seen at a considerable distance, sometimes ten or twelve feet, from the patient. On applying the ear or stethoscope to the tumor, the blood is found to rush into it with more or less violence, producing a peculiar thrill, or whizzing noise, synchronous with the contraction of the left ventricle of the heart. In the early stage of the disease, the swelling is soft and elastic, and may be readily emptied by pressure; by degrees, however, it becomes firm, tense, and, in a great measure, if not entirely, incompressible. In some cases, not in many, especially in those attended with great enlargement, there is considerable diminution of temperature of the affected limb, with indistinctness of the pulse at the wrist, or even entire absence of it, more or less cough, dyspnœa, and shortness of breath. Occasionally the pulse is fully as strong as in the other arm, but irregular or intermittent, losing several strokes in a minute.

In addition to these symptoms, there is always, when the swelling is large, so much displacement of the clavicle as to render it difficult, if not impossible, to distinguish the pulsation of the subclavian artery, the vessel being deeply buried behind and below the bone. In a few instances the collar-bone has been found to be considerably embedded in the tumor, or partially removed by absorption. Another symptom, which, from its frequent presence, especially in the latter stages of the disease, requires mention here, is the swelling of the affected limb. This varies in degree in different cases, often extends from the shoulder to the ends of the fingers, and is usually of an œdematous character, pitting under pressure, and becoming aggravated by the dependent position of the part. When thus affected, the muscles lose their contractile power, and the motions of the extremity are proportionably impaired, or entirely annihilated. Sometimes, again, owing to the great magnitude of the tumor, the patient is unable to approximate the limb to the side of the chest. Finally, there is another phenomenon, which, as it is almost invariably present in the latter stages of axillary aneurism, I am disposed to regard as pathognomonic. I allude to the peculiar attitude of the patient, arising from the constant inclination of the head towards the affected side, and the manner in which he supports the corresponding arm: the object of both being evidently to prevent the tension which would otherwise be caused in the tumor. Under these circumstances, too, the countenance wears an anxious and distressed appearance, and, as the system sympathises with the local affection, there is more or less derangement of the general health.

Notwithstanding that the symptoms of this disease are usually well-marked, it has sometimes happened that tumors of this description have been opened by ignorant practitioners under the belief that they were abscesses. For such stupidity no apology can be offered. Still, cases occasionally present themselves, though very rarely, in which it is extremely difficult, at first sight, to distinguish between this and other swellings in the arm-pit or subclavicular region. Enlarged lymphatic ganglions, adipous tumors, or encephaloid growths, for example, if they happen to lie along the course of the axillary artery, might have a pulsation imparted to them, and thus create some doubt in regard to their real character. Under such circumstances, the facility with which the tumor can be elevated or removed from the vessel, the absence of the peculiar whizzing sound, previously alluded to as being generally present in aneurismal disease, the slight pain and numbness in the part and in the corresponding limb, the diminution of the swelling on the application of pressure to the subclavian artery, and, above all, the history of the case, will generally be sufficient to enable the practitioner to arrive at a correct diagnosis.

In this disease the tumor may be *situated* either immediately below the clavicle, or in the axilla, properly so called. In the former case, it may not only elevate the clavicle, but extend up into the neck, beneath the bone, as far as the acromial margin of the scalene muscles. In the latter, it has been known to reach some distance down the arm, so as to compress the brachial artery and nerves, and prevent the approximation of the limb to the side.

Axillary aneurism, like the same disease in other parts of the arterial system, sometimes undergoes a *spontaneous cure.* An instance of this description is mentioned by Professor Cooper, in his Dictionary of Surgery, and another by M. Breschet of Paris, in his translation of Hodgson's work on the diseases of the arteries and veins. Such a mode of termination must, however, be extremely rare, and should be regarded merely as an exception to a general law, in conformity with which the disease in question, if not arrested by surgical means, will sooner or later destroy the patient. This fatal result may be brought about in one of several ways: first, by the rupture of the sac, from external violence, or from the impulse communicated to it by the current of the blood, followed immediately or remotely by exhausting hemorrhage; secondly, by suppuration, or ulcerative absorption, attended by similar consequences; and thirdly, by gangrene of the limb, or even of the aneurismal tumor itself.

Since, then, the common, if not invariable, tendency of axillary aneurism is to terminate fatally, *at what period after the developement of the disease should the operation of tying the subclavian artery be resorted to?* I answer, as early as possible; not a day should be lost after the nature of the lesion has been ascertained, and the patient placed in a condition to undergo it. Nothing surely can be gained by delay; on the contrary, the longer relief is withheld the greater will be the difficulty of securing the vessel, and the less the chance of rescuing the sufferer. From the numerous examples in which the subclavian artery has been tied, for the cure of this affection, it is evident that the anastomosing branches are fully adequate to carry on the circulation in the parts beyond the tumor. To such a degree, indeed, is this the case, that it has been repeatedly observed that there was no diminution of the temperature of the affected limb even immediately after the operation. From all the information, therefore, which we possess on this subject, it may be safely assumed, as a general proposition, that the subclavian should be secured at the earliest moment possible.

Having, as I trust, satisfactorily established the point as to the propriety of an early operation, the next question that presents itself is, *under what circumstances ought the deligation of the artery be declined?* Indiscriminate operations cannot be too much condemned, as being injurious alike to the patient, the reputation of the surgeon, and the interests of science. Like a prudent general, the surgeon should know when to retreat as well as when to advance. It is difficult to conceive of any thing more laudable than a bold undertaking in a case which must prove fatal without speedy relief. At the same time it would certainly indicate a degree of weakness, if not of downright folly, to attempt an operation where there is not the slightest prospect of a favorable issue. The circumstances in which, according to my judgment, interference would be unjustifiable, relate not merely to the tumor, but also, and that in an especial manner, to the patient himself. Enormous size of the aneurism alone is not a sufficient reason for declining an operation, since several of the examples above analyzed afford abundant proof that it may turn out well, even where every thing is apparently of the most desperate nature. In case vi, in which the aneurism was of the volume of a child's head, the subclavian artery was tied successfully by Dupuytren; and in case viii, where the size of the tumor was scarcely less, the result was equally fortunate. It is only, in short, where the lesion under consideration is combined with a worn-out state of the patient, a diseased condition of the great vessels, or some serious structural derangement of the thoracic or abdominal viscera, that an operation should be rejected as inadmissible. Under all other circumstances, no matter what may be the volume of the tumor, the duration of the malady, or the causes which have induced it, it will be our duty to afford the patient a chance for his life by cutting off the supply of blood from the aneurismal sac, and directing it into other channels, especially when that chance is the only one of which, in the present state of the science, we can avail ourselves. Even when gangrene has already commenced in the part, the surgeon should not decline the operation, provided there is nothing else to forbid it. The records of the profession afford abundant evidence of the propriety of this advice, not direct, it is true, but analogical. Thus in two cases of inguinal aneurism affected with gangrene, Sir Astley Cooper tied the external iliac artery, and though the tumors burst, no hemorrhage ensued. The coagula were discharged, the sac granulated, and the sores gradually healed.* Where the case is utterly hopeless, some advantage may be derived from strict attention to diet, confinement in the semi-erect position, gentle purgation, the repeated employment of the lancet, the occasional exhibition of an anodyne, and cold applications to the tumor. At all events, such a course would have a tendency, in some degree, to allay the patient's sufferings, and thus smooth his passage to the grave.

In the earlier attempts that were made for the radical cure of axillary aneurism, by tying the subclavian artery immediately external to the scalene muscle, great doubt appears to have been entertained in regard to the competency of the *anastomosing branches* to carry on the circulation in the parts beyond the seat of the disease. These apprehensions, however, were soon dissipated; for it was satisfactorily ascertain-

*Cooper's Surgical Dictionary, p. 102.

ed that in those cases in which the operation terminated unfavorably—and no other result was witnessed until 1817, when the vessel in question was first successfully ligatured by Dr. Post—the failure proceeded from causes entirely unconnected with the nourishment of the limb. The arteries which are more particularly concerned in maintaining the collateral circulation are, the supra-scapular, the transverse cervical, the internal mammary, the long thoracic, and the infra-scapular, the first three being branches of the subclavian, the other two of the axillary. The thoracic artery frequently arises from the aneurismal sac, and is therefore liable to be obliterated for some distance in the progress of the disease. Where the patient survives the operation for several years these vessels acquire a very large caliber, and thus compensate very fully for the loss of the parent trunk. In case xi, that of John Vaughan, who died twelve years after the subclavian was tied by Mr. Key, some of the collateral channels were dilated to three times their natural size, and so tortuous as to bear no slight resemblance to varicose veins.*

I now proceed to make some remarks on the *best method of exposing the subclavian artery*, as it emerges from behind the anterior scalene muscle. By a reference to the different cases on record it will be found that the operation has varied in the hands of different surgeons, some prefering one, others two incisions. Mr. Liston, as appears from his treatise on operative surgery,† is still an advocate for the latter proceeding, having resorted to it, I believe, in all his cases. My distinguished friend Professor Smith, of Baltimore, adopts a similar course, advising that one incision should be made along the border of the clavicle, the other co-incident with the outer

*Guy's Hospital Reports. vol. i, p. 59. 1836.
†Second Edition. p. 177. London, 1838.

border of the mastoid muscle.* Professor Gibson, of Philadelphia, recommends a horizontal incision *above* the collar-bone, commencing near the sterno-clavicular articulation, and terminating at the anterior edge of the trapezius.† My friend Dr. Mott, in one of his operations, that on William Hines, made an *oblique* incision, two inches in length, corresponding to a middle line of the triangular interval formed by the scalene muscle internally, by the clavicle inferiorly, and by the omo-hyoid muscle externally.

That two incisions, as recommended by Mr. Liston and others, are highly proper in some instances, cannot, I think, be doubted; but that they are generally advisable is what I do not believe. Where the tumor is so bulky as to produce great malposition of the shoulder, forcing the collar-bone far up into the neck, the course pointed out by the English surgeon might be followed with great advantage, as it is calculated to afford the operator much more room than it would otherwise be practicable to obtain. In all other cases, however, a single incision will, I am satisfied, be amply sufficient.

Previously to commencing the operation, the patient should be placed in the horizontal position, upon a narrow table, with his head and chest moderately elevated, and the face slightly inclined towards the sound side. An assistant taking hold of the hand, keeps the affected limb close to the trunk, at the same time that he pulls down the shoulder as much as possible, in order to draw the clavicle away, as it were, from the subclavian artery as it passes from the scalene muscle towards the first rib. The surgeon standing by the side of the patient, above the shoulder, stretches the integuments of the neck upon the upper part of the chest with the fingers of the

*Surgical Anat. of the Arteries, p, 57. First Edition. Baltimore, 1832

†Institutes of Surgery, vol ii, p. 309.

left hand, while with the other he makes an incision, about two inches and a half in length, directly along the middle of the clavicle, commencing at the sternal origin of the mastoid muscle and terminating near the anterior margin of the trapezius. In this manner he should divide the skin, the superficial fascia, and the platysma-myoid. Letting go his hold with his left hand the parts will be found instantly to resume their natural situation, leaving thus the incision on a level with the superior border of the clavicle. The next step of the operation consists in detaching from this bone the deep cervical fascia, or rather aponeurosis, which may be readily accomplished by a few gentle strokes of the handle of the scalpel instead of the point of the instrument, which would greatly endanger the surrounding vessels and nerves. The external jugular vein will be found close to the outer edge of the mastoid muscle, and should be held out of the way with a blunt hook. The supra-scapular artery will also generally appear at this stage of the proceeding, just above and behind the clavicle, or even entirely concealed by it, and should be treated in a similar manner; or, if it be divided, it should be immediately tied. The omo-hyoid lies at the outside of the incision, bound down by a process of the cervical aponeurosis, which should next be torn through with the knife. Taking this muscle for his guide, the operator feels for the tubercle of the first rib, a little to the outside of which the artery will be found pulsating, more or less distinctly, and where it may in general be easily secured by passing the needle from before backwards and from below upwards. Before tightening the ligature, it should be ascertained that it controls the circulation of the aneurismal sac, and above all that it does not include any of the cords of the axillary plexus of nerves; a circumstance which has happened in several of the cases in which this operation has been performed. It should be stated here that whenever the clavicular origin of the mastoid muscle is unusually broad, as it not unfrequently is, it should be divided upon a grooved director. By pursuing this course I am convinced that our approach to the vessel will, in the majority of cases, be greatly facilitated. Particularly will this be the case when the design is to apply the ligature *behind* the scalene muscle.

In this manner the subclavian may, in the generality of cases, be easily reached without much waste of blood, or loss of time. In some instances, however, the operation is rendered extremely difficult, tedious, and embarrassing, owing either to the magnitude of the tumor and the consequent elevation of the clavicle, the diseased state of the artery, an unusual course of the vessels of the neck, or even when these follow their natural direction, the swollen and distended condition of the subclavian vein, some irregularity on the part of the omo-hyoid, an enlarged condition of the cervical lymphatic ganglions, or, finally, the great condensation of the parts from the effusion of plastic lymph. A few remarks on each of these topics must suffice.

1. The *elevation of the clavicle* is generally in direct proportion to the size of the tumor. Sometimes, however, even where the swelling is comparatively diminutive, this bone is much higher up than usual, owing to the peculiar conformation of the individual. In either case the difficulty of finding the vessel will be much increased. In one instance, where the elevation was produced by the enormous developement of the disease, Sir Astley Cooper was forced to abandon the operation altogether. When such a contingency arises, which, on the whole, must be extremely rare, instead of giving up the case in despair, as was done by the English surgeon, the best plan, it seems to me, would be to divide the anterior scalene muscle, so as to enable us to apply the ligature somewhat nearer the heart, or even on the tracheal side of that structure.

2. The operation is occasionally rendered more or less intricate and embarrassing by the *diseased state of the artery.* In this case, although the vessel may be easily enough approached, it is impossible to apply the ligature at the usual situation. To divide the scalene muscle, or to cut down along its inner margin, for the purpose of securing the vessel in one or other of these points, is here our only resource. The brachial plexus of nerves may also be so much in the way of the operator as to render this course necessary, as happened on one occasion to Dupuytren.

3. The *cervical vessels,* both arterial and venous, are almost constantly in the way of the operator, even when they follow their usual route. This is particularly true of the external jugular vein and of the supra-scapular artery. The former of these vessels, commencing at the angle of the jaw, passes vertically down the neck, under cover of the platysma-myoid. In the early stage of its course it rests upon the sterno-mastoid, afterwards it gets to the outer border of this muscle, and finally, at the inferior part of the neck, sinks behind it, to terminate in the subclavian vein near its junction with the internal jugular. Just before it disembogues, which it sometimes does by several distinct trunks, it receives two pretty large branches, the supra-scapular and transverse cervical, which traverse the neck in a horizontal direction from without inwards, parallel with the arteries of that name. These arteries, as well as their accompanying veins, lie deeply at the root of the neck, especially the supra-scapular, which is frequently concealed for some distance by the clavicle, on a line with which it runs to reach the root of the coracoid process: it is generally a branch of the thyroid axis, and rests at first upon the anterior scalene muscle, crossing as it passes outwards the subclavian artery. The course of the transverse cervical artery is nearly similar; but it is usually situated somewhat higher up; it is also considerably smaller, and is most frequently derived immediately from the subclavian.

Now, in attempting to reach the subclavian, it is almost impossible to avoid wounding some of the vessels above mentioned. The external jugular vein is particularly in danger, and can scarcely escape without the utmost coolness and dexterity on the part of the operator. As soon as it is recognized it should be separated from the surrounding structures by a few gentle strokes with the handle of the scalpel, and drawn to the outer side of the wound. This plan is undoubtedly always the best and safest when it can be adopted; however, it sometimes happens that the vessel is so much in our way as not only greatly to embarrass our progress, but absolutely put a stop to it. In this case it must be cut across, or, what is preferable, tied with two fine ligatures, and divided between them. By pursuing this method we not only avoid a troublesome hemorrhage, but effectually prevent the introduction of air into the lower portion of the vessel, an occurrence which should be always guarded against. The supra-scapular artery, if in the way, should be carefully drawn aside, or, if it be wounded, immediately secured. Any bleeding vessels, indeed, no matter whether arterial or venous, provided they pour out a sufficient amount of blood to interfere with the operation, should at once be tied; though no more ligatures should be retained than are absolutely necessary.

4. The inordinate *swelling of the subclavian vein* is another source of embarrassment which is occasionally experienced in operations of this kind. This vessel is usually situated somewhat below and superficially to the artery, being separated from it by the anterior scalene muscle, upon which it lies. Commencing at the inferior margin of the first rib, where it is continuous with the axillary, it passes horizontally inwards, until it joins the internal jugular vein, within a few lines of the sterno-clavicular articulation. In this course, in which it is almost entirely concealed by the clavicle, it receives the small branches which accompany the different offsets of the artery, as well as the external jugular, which last enters it, as before stated, nearly opposite the centre of the bone, but sometimes more internally. After the division of the cervical aponeurosis in the lower part of the neck, the operator will occasionally observe this vessel alternately to swell and subside, owing not so much, as some have supposed, to the natural flow of the current within it, as to the reflux caused by the action of the right auricle, aided by the hurried and agitated state of the respiratory movements. The difficulty thus occasioned is not only annoying, but sometimes so embarrassing as to render it almost impossible even to see the artery, much less to separate and tie it. To remedy this it has been suggested that the operation should be suspended for a moment, and the patient placed in the semi-erect posture, to allow him to make several full and easy inspirations, after which, it is said, the tension of the vein will be diminished, and the regurgitation of the blood cease. The surest and most expeditious plan, however, that can be adopted is to hold the vessel out of the way by means of a broad blunt hook, or copper spatula, carried down behind the clavicle. In this manner the vein may be effectually compressed to the extent of half an inch, or more, if necessary, and the artery fairly brought into view.

5. The *omo-hyoid* muscle, instead of forming a triangular space, as it usually does, with the scalene muscle and the clavicle, may run parallel with, and just above, that bone, or even entirely below it. In either case, should it be productive of inconvenience to the operator, he should pass a director under and divide it. Such a proceeding, however, can seldom be called for, as by laying open its sheath the muscle may generally be drawn out of our reach. Two cases in which it became necessary to divide this muscle, before the artery could be exposed, have been reported, the one by Mr. Todd,* of Dublin, the other by Mr. Crossing,† of Davenport, England.

6. The *lymphatic ganglions* at the inferior part of the neck may be so much enlarged as to interfere materially with the different stages of the operation, as happened in a few of the cases of which I have given an abstract. When this state is found to exist, instead of trying to save these bodies, they should be carefully dissected out, as we will thus not only expedite our arrival at the artery, but, what is a matter of no little importance, greatly facilitate the healing of the parts after the vessel is tied.

7. Finally, considerable embarrassment may arise from the *condensed and indurated* state of the parts, caused by the effusion of plastic lymph. This may always be looked for when the disease is of long standing, or the tumor so large as to excite severe inflammation in the deep-seated structures immediately above the clavicle. From this cause the nerves and vessels may be not a little obscured.

If, from the above causes, it be sometimes difficult to denude the artery, to convey the ligature around it will often be found to be much more so. Indeed, this generally constitutes the most annoying and embarrassing step of the opera-

*Dublin Hospital Reports, vol. iii.
†Medico-Chirurgical Trans. vol. xvi, part 2.

tion. To facilitate this procedure various mechanical contrivances have been resorted to, some of them so complicated in their character as to be well calculated to enhance instead of diminishing the difficulty. Under ordinary circumstances every indication may be fulfilled with the common aneurismal-needle; but where the obstacles are unusually great, I know of no better instrument for successfully surmounting them than that of Mr. L'Estrange of Dublin. This consists of a steel needle, somewhat curved but not so much as common, provided with two small eyes, one behind the other, and intended to be united by a fine screw to a narrow cylindrical shaft, about three inches and a half in length, and furnished with a short ivory handle. The posterior eye being armed with the ligature, the needle is conveyed under the artery, and as soon as it appears in front of it, a small hook, passed to the bottom of the wound, is hitched into the anterior eye, when the shaft of the instrument is unscrewed, and the needle drawn round the vessel, which will thus be encircled by the thread. In case this should fail, or be not at hand, the *American* instrument as it is termed, invented by Dr. Parrish of Philadelphia, may be advantageously resorted to. The aneurismal-needle, devised and recommended by Dr. Gibson, is so awkward and clumsy, as it is usually constructed, that it should be altogether discarded from practice. Whatever mechanical contrivance be employed, the surgeon should always proceed, in this stage of the operation, in the most cautious and gentle manner, taking care not to separate the artery unduly from its cellular connexions, or to inflict the slightest violence upon its coats. The ligature, as a general rule, should be passed from before backwards and from below upwards, as it will be found much easier in this way to prevent injury to the subclavian vein, while there will be no danger whatever of including any of the cords of the brachial plexus of nerves. Owing to the great depth of the wound, great difficulty is sometimes experienced in tightening the knot. In this case the ingenious instrument described by Mr. Liston, in the sixteenth volume of the Edinburgh Medical and Surgical Journal, may be resorted to. That invented by Dr. Hosack, of New York, will also be found very useful. These contrivances, by holding the first knot firm, enable the surgeon to tie a second or third with the utmost facility.

Notwithstanding the assistance to be derived from the different instruments that have been invented for that purpose, such has been the difficulty, in some instances, of conveying the ligature around the artery as to lead not only to great delay, but almost to an abandonment of the operation. It has been suggested, under these circumstances, to saw through the clavicle. To this proposal, which originated, I believe, with Mr. Hargrave, an Irish surgeon, I can see no particular objection, provided the shoulder is so much elevated as to offer an almost insurmountable barrier to the passage of the ligature. By this practice, although a compound fracture of the clavicle would be superadded, yet this would be of the simplest kind, while the operation, instead of occupying from one to two hours, as has repeatedly happened heretofore, could be completed in a comparatively short time ; the vessel could be much more effectually secured, the risk of wounding the subclavian vein and other parts would be greatly diminished, and the patient would have a much better chance for recovery. It is proper to remark, however, that this proposal has been pointedly condemned by some very eminent surgeons, as being both dangerous and uncalled for, and that it has not, so far as my knowledge extends, been hitherto carried into execution.

It will be perceived that the foregoing observations relate exclusively to the subclavian artery in its third stage, or to that portion of it which lies between the inferior border of the clavicle, where it is continuous with the axillary, and the acromial margin of the anterior scalene muscle. It has also been remarked, incidentally, that the vessel has been tied in several instances *behind* that muscle; and it may now be added, in conclusion, that it has been secured not less than three times—perhaps oftener—though unsuccessfully—in the first part of its course, or internal to the scalene muscle. All these operations were performed on the right side, and, what is singular, by Dublin surgeons; first by Mr. Colles in October, 1811; secondly by Mr. Hayden in September, 1835; and thirdly by Mr. O'Reily in April, 1836.*

In Mr. Colles' case the ligature was not tightened until the fourth day from the operation, owing to the occurrence of great dyspnœa and cardiac oppression. On the ninth day the patient, who was a laborer, thirty-three years of age, complained of a sense of strangling and pain about the heart; he then became delirious, and died in a few hours. Not above a quarter of an inch of the subclavian was found free from disease, and on this part the ligature had been applied. Immediately above this point a pretty large opening had been made through the coats of the vessel by ulceration. The tumor, which was of a conical form, reached from the sternal origin of the mastoid muscle beyond the arch of the clavicle, rising nearly two inches above this bone. The operation is

*Since writing the above I learn, by reference to the Philadelphia Medical Examiner for May, that Mr. Partridge, one of the surgeons of King's College Hospital, London, tied the subclavian artery in the first part of its course, for an aneurism which had existed in the vessel for about twelve months. The pulsation in the tumor immediately ceased, and, although every thing went on favorably for a short time, death occurred on the fourth day after the operation.

described as having been exceedingly tedious, on account of the difficulty of passing the ligature.*

In the case operated on by Mr. Hayden death occurred on the twelfth day, preceded by bronchitis and hemorrhage from the wound. The subclavian artery, at the site of the ligature, was gaping irregularly for three-fourths of its caliber, the remainder being sound. The right carotid was considerably enlarged, the surrounding parts were all agglutinated by plastic lymph, and the tumor extended to the anterior scalene muscle, beneath the clavicle, and between the branches of the brachial plexus of nerves. Its contents were throughout solid and laminated. A small aneurism was found on the axillary artery. No attempt at restoration could be discovered. The patient was an unmarried woman, of intemperate habits, fifty-seven years of age. The disease had existed about ten months.†

The subject of Mr. O'Reily's case was a stout robust man, thirty-nine years of age, for the last twenty years of which he has been employed as a stable servant. The aneurismal tumor, which had been first observed the preceding February, was somewhat of an oval shape, distinctly circumscribed, and measured two inches and a half in one direction by two inches in the other. The operation was performed on the sixteenth of April; hemorrhage occurred on the fourteenth day; on the twenty-second day the patient had troublesome cough, with a return of the bleeding; and the next day, the twenty-third, he expired. On opening the body at least twenty ounces of grumous blood were found in the wound, along the trachea, and between the spine and the apex of the right lung, which was slightly hepatized. The pleura and

*Edingb. Med. and Surg. Journal, vol. ii. January, 1815.

†Lancet, Oct, 7, 1837.

bronchia were also inflamed, the carotid thickened and enlarged, and the divided extremities of the subclavian, jagged and irregular, were patulous and separated nearly two inches by coagula. Not the slightest attempt had been made at reparation. The aneurismal tumor, four inches in length, extended from the acromial margin of the scalene muscle to the commencement of the axillary artery, which had a similar swelling, about an inch in diameter, connected with it. The operation occupied twenty-five minutes.*

It is worthy of remark that in two of the above cases the patients were affected with *acute bronchitis*, brought on, in all probability, by the injury inflicted upon the parts in the immediate vicinity of the right lung. What the precise condition of that organ was in the individual operated on by Mr. Colles, is not known, as the post mortem inspection seems to have been very hasty and imperfect. In neither of the cases had there been any attempt at the reparative process, although in one of them, that of Mr. O'Reily, life was protracted until the twenty-third day after the operation; the artery in all was in a state of ulceration, and in two of them death was preceded by hemorrhage from the wound. So far, therefore, as these examples go, they hold out no inducement whatever for a repetition of the operation. Nevertheless, there is no reason why we should despair; for, as has been very justly observed by Dr. Colles, who was the first to tie the vessel in this situation, the history of surgery furnishes parallel instances of proceedings, now generally adopted, which, in the few first instances, failed of success. Mr. Hayden of Dublin, under a belief, in all probability well founded, that the impetus of the blood, in consequence of the close proximity of the heart, has a great share in disturbing the sanative process set

*Flood on the Surgical Anatomy of the Arteries, p. 74. London: 1837.

up by nature at the site of the ligature, suggests the propriety of securing both the common carotid and subclavian at the same time. A coagulum, he thinks, would thus soon form, which would close up these vessels on the cardiac side of the ligature, as well as the whole arteria innominata. Although this ingenious suggestion has not yet been acted upon, it is certainly worthy of consideration, emanating, as it does, from an able and distinguished surgeon.

I shall close this paper, already extended greatly beyond what was originally intended, with an account of the operation of tying the subclavian artery, in the first part of its course, as performed by Mr. O'Reily, of Dublin.

Standing by the patient's right side, whose head was slightly depressed and turned to the left, Mr. O'Reily first drew down the common integuments of the inferior part of the neck over the clavicle with his left hand, and then cut freely on the bone, beginning his incision about the centre of the clavicular origin of the right mastoid muscle, and extending it across the trachea as far as the centre of the sternal attachment of the left mastoid muscle; the whole being about four inches in length. Another incision was next made through the skin along the internal margin of the right mastoid, so as to meet the preceding at its middle: in the same line he divided successively on a director the superficial fascia and platysma-myoid. The sternal origin, with the inner-half of the clavicular, of the right mastoid muscle was divided transversely close to the bone, and detached. On introducing a finger the course of the carotid artery could be distinctly seen and felt. The deep aponeurosis, together with a little of the internal margins of the sterno-hyoid and sterno-thyroid muscles, was next divided, so as to expose the carotid, the sheath of which was pinched up with a pair of forceps, and cautiously opened horizontally. A blunt silver instrument, of the size of a small scalpel, with a round point, was used in the subsequent steps of the operation. The carotid being taken as a guide, the subclavian artery was easily exposed lying at the bottom of a very deep cavity. The jugular vein was drawn outwards by means of a curved spatula, and the pneumo-gastric nerve inwards. The artery was then secured with L'Estrange's needle, described in a preceding page, passed from below upwards, the ligature tightened, and the wound closed with two adhesive straps.*

On the left side the vessel is so deeply seated and in such intimate relation with the bag of the pleura, the carotid artery, the internal jugular vein, the pneumo-gastric nerve, and the thoracic duct, that the deligation of it must be considered as altogether impracticable. At all events no attempt of the kind has yet been made, nor is it likely that, if it should be, it would prove successful. From the severe injury that would necessarily be inflicted upon the surrounding structures, during the performance of the operation, violent and fatal inflammation would be the inevitable and speedy consequence.

It is my intention, in a future number of the journal, to make some remarks on aneurism of the carotid, iliac, femoral, and popliteal arteries, considering the subject in reference to the age of the patient, the causes and duration of the disease, the effects of treatment, both palliative and radical, and various other circumstances, which it is unnecessary here to specify.

---

*Flood, op. cit., p. 80.

