# Erratum

**Published:** 1841-06

**Authors:** 


ERRATUM.—Page 416, line 20, for twenty-seven read twenty-six.

